# A Rare Case of Elevated Transaminases With Incomplete Abortion Due to Cytomegalovirus Infection: An Experience From a Resource-Limited Setting

**DOI:** 10.7759/cureus.41331

**Published:** 2023-07-03

**Authors:** Suman K Jha, Bhesh R Karki, Sudeep Yadav, Bibek Karna, Ranjit B Jasaraj

**Affiliations:** 1 Internal Medicine, University of Louisville, Louisville, USA; 2 Internal Medicine, Downstate-Health Sciences University, New York, USA; 3 Rheumatology, The University of Chicago Medicine, Chicago, USA; 4 Internal Medicine, Lower Bucks Hospital, Bristol, USA; 5 Internal Medicine, Mount Sinai Hospital Medical Center of Chicago, Chicago, USA

**Keywords:** elevated liver enzyme, pregnancy, incomplete abortion, transaminitis, cytomegalo virus (cmv)

## Abstract

Cytomegalovirus (CMV) infection during pregnancy may cause spontaneous abortion, stillbirth, and death of newborns. CMV is the most common congenital infection in newborns. It generally has a benign course in immunocompetent individuals, while the severe disease is usually seen in immunocompromised patients. Most of the published studies about CMV infection describe congenital abnormalities in newborns. Only a handful of case reports mention CMV infection associated with elevated transaminases during pregnancy. Here, we present a case of incomplete abortion with elevated liver enzymes in a 26-year-old female caused by CMV infection. Our case report illustrates the importance of considering CMV infection as a differential in an incomplete abortion associated with elevated liver enzymes.

## Introduction

Cytomegalovirus (CMV) is the leading infectious cause of congenital infection in newborns, affecting three to six per 1,000 live-born infants each year in the United States [1]. CMV infection in immunocompetent individuals has an asymptomatic course or may present with a mononucleosis-like syndrome (fever, hepatosplenomegaly, pharyngitis, and lymphadenopathy with a negative monospot test) [2]. In a study, 31% of pregnant women who acquired primary CMV infection experienced fever, myalgia, and flu-like symptoms, which were evident in our case [3]. In patients with symptomatic CMV infection, a liver function test is frequently deranged, and subclinical elevated transaminases are the most common finding. The severity may range from mildly raised liver enzymes to acute liver failure [4,5]. In this case report, we describe a rare case of CMV infection with elevated transaminases, leading to an incomplete abortion.

## Case presentation

A 26-year-old primigravida female at seven weeks of gestation presented to the emergency department with a chief complaint of per vaginal (PV) bleeding for a five-hour duration. It was associated with lower abdominal pain for the same duration. The PV bleeding was sudden in onset with the passage of clots, bright red in color, and soaked about two pads. She reported lower abdominal pain that was dull aching and non-radiating. She had an episode of subjective fever (not recorded) and malaise three days prior to the presentation for which she did not t seek any medical advice. She denied sore throat, recent drug use, loss of consciousness, bleeding from any other sites, recent sick contacts, and recent trauma.

On admission, she had a temperature of 97 F, pulse rate of 88 beats/minute, respiratory rate of 20 breaths/minute, and blood pressure of 130/90 mm of Hg. Her oxygen saturation was 96% in room air. Mild pallor and icterus were present. Physical examination revealed a soft abdomen with mild tenderness in the hypogastric region without rebound tenderness and organomegaly. Chest and cardiovascular examinations were within normal limits. Per speculum examination showed blood clots with active bleeding. PV examination revealed a six-week-size uterus, open cervical os, and free and non-tender bilateral fornices suggestive of incomplete abortion.

Laboratory results revealed hemoglobin of 11 mg/dL, raised white blood cell count of 15,800 cells/mL of blood with 40% neutrophils, 55% lymphocytes, and 5% monocytes. She had slightly raised urea of 28.6 mg/dL and normal creatinine of 0.9 mg/dL. Her liver function test was deranged with alanine aminotransferase (ALT) at 73 IU/mL, aspartate aminotransferase (AST) at 256 IU/mL, alkaline phosphatase at 745 IU/mL, total bilirubin at 6.5 mg/dL, and direct bilirubin at 1 mg/dL. The mean corpuscular volume, reticulocyte count, and Coomb’s test were normal. Her bleeding time, clotting time, and prothrombin time/international normalized ratio were within normal limits. Beta-human chorionic gonadotropin (beta-hCG) was 4,650 mIU/mL (Table 1). Her abdominal ultrasound revealed minimal free fluid in the right upper quadrant to the right lower quadrant, normal liver/gallbladder, irregularly thickened and inhomogeneous endometrium, prominent vasculature, and retained products of conception in the uterus suggestive of incomplete abortion. The hepatitis panel for A, B, and C and human immunodeficiency virus (HIV) tests were negative. Serology for CMV, urine, and blood culture was sent.

**Table 1:** Laboratory values from admission to discharge and follow-up.

Laboratory Value	Reference Range	Day 1	Day 3	Day 5	Follow-Up (Day 12)
Aspartate aminotransferase (IU/L)	10-40	256	240	151	26
Alanine aminotransferase (IU/L)	10-40	73	73	83	21
Alkaline phosphatase (IU/L)	40-112	745	491	348	43
Total bilirubin (mg/dL)	0-1	6.5	5.3	2.5	1.1
Direct bilirubin (mg/dL)	0-0.35	1.0	1.3	0.3	0.2
WBC (x10^9^/L)	4.5-11.0	15,800	13,200	9,700	8,500
Neutrophils %	40-80	40	43	55	65
Lymphocyte %	20-40	55	48	40	25
Beta-hCG (mIU/mL)	4,059-153,767	4,650	2,120	720	14

The patient was treated with intravenous fluids (normal saline bolus of 1,000 mL, followed by 150 mL/hour for 24 hours) and sublingual misoprostol 800 mcg in the emergency department. She was then shifted to the medical unit for further observation and management. During her stay in the unit, she had spikes of fever for the first two days, which was treated with acetaminophen. Her serology resulted positive for CMV IgM antibody at 50 U/mL detected by the enzyme-linked immunosorbent assay (ELISA) test kit. However, an avidity test was not performed due to its unavailability at our center. Urine and blood cultures were negative. The patient was managed conservatively, and labs were monitored on alternate days. The patient improved gradually and was discharged on the fifth day of admission. At one week follow-up after discharge, she was asymptomatic, and the repeat labs were normal (Table 1).

## Discussion

CMV is the leading cause of congenital malformation and disability in newborns in the United States [6,7]. CMV, also known as human herpes virus-5 (HHV-5), is a double-stranded linear DNA virus and a member of the family Herpesviridae. CMV usually causes an asymptomatic infection; thereafter, it remains latent in the body and reactivates intermittently. CMV virus is transmitted via saliva, urine, sexual contact, blood transfusion, organ transplantation, and placenta (congenital CMV infection) [8,9]. The incidence of primary CMV infection in pregnancy is higher in lower socioeconomic groups [10,11]. Thus, there is an increase in the burden of congenital infection and malformation among newborns in resource-poor countries.

The symptoms of CMV infection vary depending on the age and immune status of a patient. The incubation period of CMV is about four to six weeks. Immunocompetent individuals generally follow an asymptomatic course; however, it can involve any organ system. The most affected organs include the gastrointestinal tract (gastroenteritis, colitis, transaminitis), followed by the central nervous system (retinitis) [12,13]. Subclinical elevated transaminases are the most common manifestation, while cholestasis and portal vein thrombosis are less typical [14]. However, lab results in our patient suggested elevated transaminases and cholestasis (elevated bilirubin and alkaline phosphatase). A deranged liver function test for CMV infection is likely the result of viremia. The prognosis of CMV transaminitis in an immunocompetent patient is generally reassuring and requires no treatment. Our patient responded well to conservative management.

Lymphocytosis, especially when seen with elevated liver enzymes and in an appropriate clinical context, can be one of the earliest indicators for CMV infection during pregnancy, as seen in this patient. The most common serologic test to detect CMV antibodies (IgM and IgG) is the ELISA test, which was used at our center [15]. An anti-CMV IgG avidity test diagnoses primary CMV infection in pregnant women. This test helps distinguish primary CMV infection from past infection. Low titers of IgG suggest recent infection (within two to four months), while high titers suggest past infection [16]. The quantitative polymerase chain reaction (PCR) test is highly sensitive and is the standard test for diagnosing CMV infection [17]. However, due to the unavailability of the PCR test at our center, CMV PCR could not be done in this patient.

Abortion resulting from CMV infection is an under-explored topic in research studies. In a retrospective cohort study of 84,699 women from Israel, the incidence of primary CMV infection was 14.5 per 1,000 [18]. Another study conducted on 340 cases showed a high frequency of CMV antigens in tissues from first-trimester abortion [19]. CMV infection should be screened during pregnancy, especially in patients with fever, malaise, and elevated liver enzymes, to prevent abortion and congenital malformation in newborns. 

Ganciclovir is the preferred medication for treating severe manifestations of CMV infection, such as CMV retinitis. It is also used for prophylaxis against CMV infection in transplant recipients. However, no treatment has been approved for CMV infection in pregnancy. Few studies show the effectiveness of valacyclovir and hyper-immune globulin (HIG) in pregnant females for the prevention and treatment of congenital CMV infection, but we need a large cohort of patients to generate high-quality evidence [20]. Immunocompetent pregnant patients with CMV infection are generally managed with supportive care.

## Conclusions

Abortion is a rare complication of CMV infection. Clinicians should suspect CMV infection as one of the differentials in a pregnant female with elevated liver enzymes after ruling out common etiologies, such as hepatitis A/B/C, alcoholic hepatitis, non-alcoholic fatty liver disease, and drug-induced liver injury. CMV infection should be screened during pregnancy, especially in patients with fever, malaise, and elevated liver enzymes to prevent abortion and congenital malformation in newborns.
